# Host population diversity as a driver of viral infection cycle in wild populations of green sulfur bacteria with long standing virus-host interactions

**DOI:** 10.1038/s41396-020-00870-1

**Published:** 2021-01-15

**Authors:** Maureen Berg, Danielle Goudeau, Charles Olmsted, Katherine D. McMahon, Senay Yitbarek, Jennifer L. Thweatt, Donald A. Bryant, Emiley A. Eloe-Fadrosh, Rex R. Malmstrom, Simon Roux

**Affiliations:** 1 Joint Genome Institute, Berkeley, CA USA; 2 University of Wisconsin, Madison, WI USA; 3 University of California Berkeley, Berkeley, CA USA; 4 The Pennsylvania State University, University Park, PA USA

**Keywords:** Bacteriophages, Metagenomics

## Abstract

Temperate phages are viruses of bacteria that can establish two types of infection: a lysogenic infection in which the virus replicates with the host cell without producing virions, and a lytic infection where the host cell is eventually destroyed, and new virions are released. While both lytic and lysogenic infections are routinely observed in the environment, the ecological and evolutionary processes regulating these viral dynamics are still not well understood, especially for uncultivated virus-host pairs. Here, we characterized the long-term dynamics of uncultivated viruses infecting green sulfur bacteria (GSB) in a model freshwater lake (Trout Bog Lake, TBL). As no GSB virus has been formally described yet, we first used two complementary approaches to identify new GSB viruses from TBL; one in vitro based on flow cytometry cell sorting, the other in silico based on CRISPR spacer sequences. We then took advantage of existing TBL metagenomes covering the 2005–2018 period to examine the interactions between GSB and their viruses across years and seasons. From our data, GSB populations in TBL were constantly associated with at least 2-8 viruses each, including both lytic and temperate phages. The dominant GSB population in particular was consistently associated with two prophages with a nearly 100% infection rate for >10 years. We illustrate with a theoretical model that such an interaction can be stable given a low, but persistent, level of prophage induction in low-diversity host populations. Overall, our data suggest that lytic and lysogenic viruses can readily co-infect the same host population, and that host strain-level diversity might be an important factor controlling virus-host dynamics including lytic/lysogeny switch.

## Introduction

Recent advances in metagenome sequencing have enabled high-throughput exploration of the virosphere, leading to a >200-fold increase in viral genomes available in databases, and uncovering >1,000 new genus-level viral groups [1–4]. While these data provided numerous new insights into viral genome diversity, most of these uncultivated viral genomes are not associated with any host [5]. The scarcity of host association for uncultivated viruses means that basic viral ecology parameters, such as how many different viruses infect a single host population and how the diversity of virus and host populations change over time, are poorly understood. Correspondingly, this limits our ability to fully understand virus-host interactions in nature, and severely hinders efforts to integrate viruses into ecosystem models.

For viruses infecting bacteria and archaea, viral infection dynamics exist along a spectrum from highly lytic to lysogenic and/or chronic [6]; highly lytic viruses undergo viral replication and host lysis immediately, while temperate viruses have a ‘latency’ period where the viral genome reside in the host cell (‘lysogenic’ infection) before replication and host lysis. Many cultivated and uncultivated phages seem to be temperate [7], however the ecological and evolutionary mechanisms affecting these different infection dynamics are still unclear. Specifically, one of the most fundamental questions in viral ecology that remains unanswered is which environments and/or life-history traits select for more lytic or temperate viruses. This is an actively researched and discussed topic, both in a viral ecology framework [8–12], and a molecular biology framework [13, 14]. Building from a combination of experimental virus-host systems and whole-community studies, different hypotheses about these ecological and evolutionary drivers have been proposed. The “kill the winner” model places importance on the lytic life stage of viruses, and proposes that when a host is actively growing, host population numbers increase, and thus lytic infections will be favored over lysogenic infections [15]. In this scenario, lytic viruses suppress host blooms, and maintain diversity in the host population by preventing one host strain from out-competing all others [9, 16]. These recurring lytic infections can significantly impact both host and virus genome evolution, including through the formation of “genomic islands” of high variability [17, 18]. When hosts are not as readily available, lysogeny is seen as an “alternative” choice in this model. Several studies similarly suggested that lysogeny may be governed by broad environmental parameters such as trophic status and nutrient availability. For example, lysogeny seems to be favored in environments where bacterial productivity is low, or for hosts with variable growth rates (“boom-and-bust” cycles), such as pathogenic microbes or environments with strong seasonal patterns [19–21]. In contrast, the “piggyback-the-winner” model proposes instead that lysogeny is favored when hosts are readily available [9, 10]. This may be advantageous in environments with rapidly growing hosts, where the viruses would profit more from lysogeny than from host lysis, for example. This scenario reduces the amount of control viruses could exert over bacterial abundance, and places more importance on other types of host interactions, e.g., superinfection exclusion conferred by the lysogens [9].

The existence of such diverging hypotheses, both with supporting data, reflects the complexity of virus-host interactions and the limits of our current understanding. In particular, the majority of the hypotheses put forward so far have been based on community-wide measurements, which average out differences between individual viruses and host populations [7, 9]. In addition, most focus on ecophysiological traits, such as host growth rate and nutrient availability, and only a few consider host and virus population diversity, although virus-host coevolution mechanisms at the strain level have been described [16, 17, 22]. In order to expand our understanding of these host–virus dynamics, high-resolution datasets that incorporate a variety of specific host–virus pairs across time and environments are required.

In this study, we focus on uncultivated viruses infecting green sulfur bacteria (GSB) in a model freshwater lake (Trout Bog Lake, TBL) sampled from 2005 to 2018 by the North Temperate Lakes Microbial Observatory. GSB are anoxygenic photoautotrophic bacteria that can form massive blooms in illuminated, low-oxygen, sulfidic waters, and play a central role in global carbon and sulfur cycling [23–25]. Because GSB are strict anaerobes and many grow slowly, direct isolation of GSB viruses in the laboratory remains challenging. As a consequence, viruses infecting GSB have yet to be formally isolated and identified, leaving their diversity and impact on environmental GSB populations still relatively unknown. Yet viruses likely influence bloom dynamics of aquatic GSB populations, and may be important agent of horizontal gene transfer (HGT), especially since comparative genomics previously revealed an extensive history of HGT across GSB [26, 27]. The predictable dynamics of GSB in TBL with strong seasonal patterns and high-density blooms forming in summer also make these an interesting model system to explore the influence of environmental parameters, host productivity, and host life-history traits on viral infection dynamics over multiple years.

To investigate viral infection dynamics of GSB in TBL, we first used two complementary approaches to identify viruses infecting GSB. We next followed newly-established virus-host pairs across multiple years and seasons using bulk metagenomes covering the 2005–2018 period to evaluate patterns of infection rate and virus-host interactions at the population level. Finally, we highlight through direct observation and theoretical modeling that host strain-level diversity may be a critical parameter driving these virus-host dynamics.

## Results and discussion

### GSB abundance and targeted recovery in TBL

**Fig. 1:**
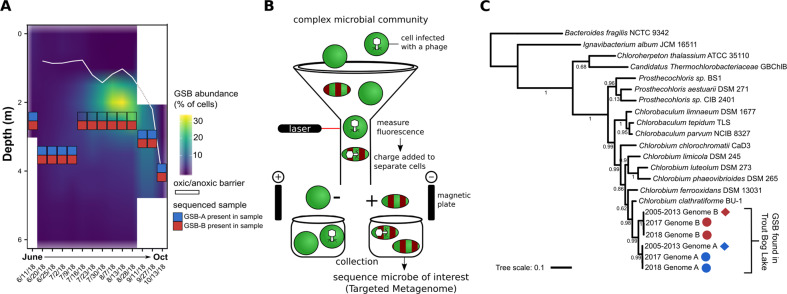
Abundance and diversity of green sulfur bacteria (GSB) in Trout Bog Lake. **A** An average of 3.7 million cells/ml were measured per sample per date; shown is GSB abundance (% of total cells per sample per date). Blue/red boxes are positioned at the specific depth from which targeted metagenomes were sequenced, with colors corresponding to the detection of GSB-A (blue) and/or GSB-B (red) in these targeted metagenomes. The white line represents the oxic/anoxic barrier. **B** GSB cells were sorted using FACS, targeting green DNA stain and red autofluorescence. **C** Phylogenetic tree based on DNA sequence of rpoB (beta subunit of RNA polymerase) genes of GSB from Trout Bog Lake and GSB isolate genomes; circles represent new genome bins from our targeted metagenomics, and diamonds represent previously published genome bins [31]; colors represent Genome B (red) and Genome A (blue).

During the summer and early fall of 2018, TBL was sampled on a nearly weekly basis to evaluated GSB abundance and generate the metagenomic data introduced in this paper. Two types of sampling were performed: one targeted GSB by sampling specific depths of the water column (depth-discrete) followed by Fluorescence Activated Cell Sorting (FACS), and the other aimed to collect the entire microbial community by sampling across the hypolimnion, i.e., the bottom layer of the lake (standard bulk metagenomes from integrated samples, see Methods and Fig. S1). Prior to processing these samples, we first verified that we could use FACS to robustly distinguish GSB from other microbial cells based on autofluorescence using an existing freshwater GSB isolate (*Chlorobaculum tepidum*, see Methods). Then, the relative abundance of GSB in TBL was quantified using FACS at individual depths (depth-discrete samples) across all sampling dates in 2018 (Fig. 1A, B). While other bacteria could potentially display fluorescence profiles similar to GSB, targeted metagenomes sequenced from cells identified as “GSB-like” by FACS were near-exclusively composed of GSB sequences (see below), supporting our use of FACS data as a measurement for GSB abundance.

Early in the summer, we measured ~140,000 GSB cells/ml, and GSB peaked in mid-August with 2.2 million GSB cells/ml (32% of total cells screened). The overwhelming majority of GSB cells were found right below the oxygen barrier/chemocline (white line in Fig. 1A), consistent with the anoxygenic photosynthetic metabolism of GSB. This type of seasonal pattern for GSB has been observed in other freshwater lake systems, including through 16S rRNA gene amplicon sequencing data [28], where GSB were dominant during the summer when the lake developed stratified layers. For all timepoints for which depth-discrete samples were available, we generated five replicates of GSB-targeted metagenomes (5,000 GSB-like cells sorted based on size and autofluorescence, see Fig. S2), along with two replicates of non-GSB metagenomes (5,000 cells positive for DNA stain, but negative for autofluorescence), from the depth at which the GSB cell count was highest. In total, targeted metagenomes were generated from 14 sampling dates in 2018, and one sampling date in fall 2017 (see Methods, Fig. S1).

### Targeted metagenomes uncover two distinct GSB populations

Bacterial genomes were binned from a combined assembly of GSB-targeted metagenomes from each sampling date, resulting in 14 sets of 2018 genome bins (one set per date). Each sampling date included 1 or 2 nearly complete GSB genome bins (completeness estimation: 97–100%, redundancy estimate: 0–2%, Table S1), with no other taxa binned in our sorted samples. Based on average nucleotide identity (ANI) clustering, bins from all 14 sampling dates in 2018 were found to represent 2 distinct genomes, hereafter designated as “GSB-A” and “GSB-B” (Table S2A). A similar approach was used to generate GSB genome bins from the 2017 depth-discrete targeted metagenomes (a single depth and sampling date, Fig. S1).

Previous exploration of GSB community diversity in lakes using methods such as amplicon sequencing and fingerprinting also typically found only a few different GSB in each location, suggesting that only a few dominant GSB populations occur in a given environment [27–30]. When placed in a phylogeny with known isolate genomes and previously published TBL genome bins, both GSB-A and GSB-B branched within the *Chlorobiaceae* family, and tightly clustered with the previously described TBL GSB genomes (Fig. 1C) [31]. ANI comparison confirmed that both GSB genome bins recovered from targeted metagenomes were the same populations as those found in the bulk metagenomes throughout 2005–2013 (ANI > 99.5%). Notably, GSB-A corresponds to a GSB population that was previously observed to undergo a genome-wide sweep between 2005 and 2009 (Chlorobium-111 in ref. [31], Table S2B, C).

Because we targeted the GSB maximum depth at each time point (to sample the depth with maximum GSB concentration), targeted metagenomes were sequenced from different depths across 2018, highlighting differential distribution patterns for GSB-A and GSB-B (see Fig. 1A). Specifically, GSB-A was only recovered at lower depths (3–4 m) (with the one exception of the June 11th sampling date, early in the summer), while GSB-B was recovered at all sequenced depths, including higher in the water column during the summer bloom (2.5–3 m, Fig. 1A, Table S1). This suggests that the co-existence of these two GSB populations in TBL is possibly linked to niche partitioning along the water column, e.g., due to local biogeochemical conditions and/or light regimes. This would be consistent with the genomic differences between GSB-A and GSB-B, which despite being classified as two closely related species, display ~23% unique gene content with respect to each other. This type of niche partitioning has been seen in GSB before, with brown-pigmented GSB blooming deeper than green-pigmented GSB [32, 33]; and this type of layering can also be found within green-pigmented GSB, depending on which specific pigments they produce [34]. Based on pigment gene analysis, both our GSB populations appear to be green-pigmented GSB, and both populations have an intact *bchU* gene, while neither have *bciD* [35] nor *cruB* genes [34]. It is unclear if they produce the same green pigments, as the same set of pigment genes can be found in both genomes, but the actual sequences of the *bchU* genes are not identical between the two GSB. In any case, further work is required to evaluate hypotheses about niche partitioning specifically in these TBL populations.

A third bin was routinely found in the 2018 targeted metagenomes, however it was estimated to be only 20% complete, i.e., it included very few to none of the expected core bacterial genes. This third bin was clustered next to the other two GSB bins when included into an rpoB phylogeny, suggesting it also represented a GSB population (Fig. S3). A similar genome was also binned in the 2017 targeted metagenomes, but never in the bulk metagenomes from 2005 to 2013 even though read mapping revealed that the corresponding sequences were present in those datasets at a low relative abundance, suggesting that this third bin could represent a distinct and rare GSB population. Considering that we never recovered a complete genome from the third bin, and that no viral contigs were found in this bin, only GSB-A, GSB-B, and their associated viruses were further analyzed.

### Distinct GSB viruses are recovered from targeted metagenomes and CRISPR matching

To identify viruses infecting GSB, we first used VirSorter to detect viral contigs from the 2017 and 2018 GSB-targeted metagenomes, and excluded putative contaminants based on coverage patterns across replicates (see Methods). Across all 14 sampling dates in 2018, we recovered 43 nonredundant predicted GSB viral contigs (clustered at 95% ANI—80% alignment fraction, AF) (Table S3). While most of these 43 viral sequences were short and could represent decayed prophages or rare viruses that might be difficult to assemble, we identified 11 complete or nearly complete GSB virus genomes, on which we focused our analysis. Out of the 11 viruses, 10 were identified as temperate (i.e., able to enter a lysogenic cycle) because they encoded an integrase gene and/or were assembled as an integrated prophage within our GSB genomes. The other viral contig, CV-1–33, did not contain an integrase gene, nor was it ever assembled as integrated into the host genome, and thus is probably not a temperate virus. This high number of prophages is in contrast to a recent search which did not detect any prophage in >80% of Chlorobi genomes, and only 1 in the remainder [20]. It remains uncertain however, if this discrepancy reflects a biological difference between TBL GSB and isolated *Chlorobium* strains, or technical biases associated with isolation or prophage identification.

Putative GSB viruses were also recruited from all available TBL metagenomes from 2005 to 2018 by matching spacers from the CRISPR arrays associated with GSB-A and GSB-B to predicted viral contigs (see Methods). This yielded a total of 534 contigs, which were subsequently clustered into viral OTUs (95% ANI and 85% AF) to produce 45 nonredundant viral contigs (34 of the contigs were >10 kb). Out of the 45 contigs, 6 matched viruses recovered through GSB-targeted metagenomes (see above). Overall, combining results from the flow sorting method (targeted metagenomes) and the CRISPR spacer-matching method resulted in 50 nonredundant GSB viruses. Only one putative GSB viral sequence was described in the past, identified as a virus infecting GSB based on metagenomic coverage, however it bears no similarity to the GSB viruses identified here, either in flow sorting of from CRISPR [27].

### Comparative genomics of GSB viruses highlight contrasting patterns of genome evolution

**Fig. 2:**
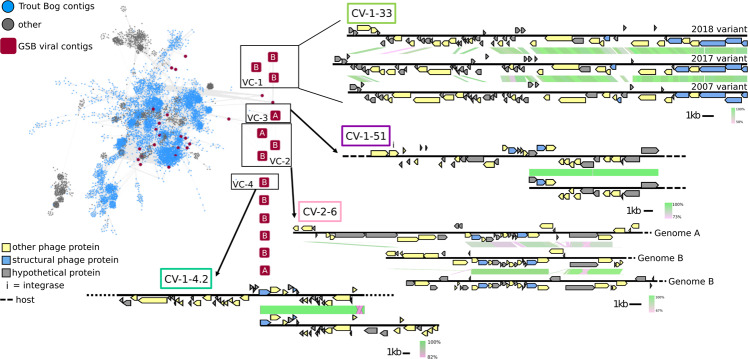
Genomic diversity of Trout Bog Lake GSB-associated viruses. Viral contigs from Trout Bog Lake (TBL) were clustered with genomes from the NCBI Viral RefSeq database [82] as well as predicted GSB viruses from IMG/VR [5] and Lake Mendota viral contigs, based on pairwise gene comparison. GSB viral contigs were dereplicated prior to clustering, so that only one copy of each genome was included in the network. Individual viral contigs are colored according to their origin (gray for sequences not from Trout Bog Lake, blue for Trout Bog Lake contigs not associated with GSB, and red for Trout Bog Lake contigs associated with GSB). The GSB-associated viral contigs detected in the GSB-targeted metagenomes are highlighted with a square shape. Network edges represent shared gene content between viral contigs. GSB viral contigs clustered into four groups (VC-1, -2, -3, and -4). VC-1 contains the three CV-1-33 variants; VC-3 and VC-4 contain CV-1-51 and CV-1-4.2, respectively, plus a previously sequenced TBL metagenome viral contig from 2008. Pairwise genome alignments are represented next to each cluster. Genome alignments were generated using blastn, with green representing 100% nucleotide alignment. Gene content is color-coded, and dashed lines show regions where the host genome was found. All GSB viral contigs from targeted metagenomes (red squares) are labeled with their host (either “A” for GSB-A or “B” for GSB-B).

To situate these new GSB viruses within the global virus diversity, we used a genome-based network analysis of their shared protein content with vContact 2. The majority of the new GSB viruses (45/50) were connected to the main component of the network, confirming that these are likely members of the *Caudovirales* order (Figs. 2, S4). However, they formed novel clusters (approximately genus/subfamily rank), which did not include any other reference sequences beyond GSB viruses. This is consistent with the lack of isolated GSB viruses, and the fact that viruses tend to cluster by host groups (~phylum or class rank) in gene-content-based networks [36]. Within the GSB viruses, most sequences tend to cluster by themselves (as singletons), with two exceptions where distinct GSB viruses were grouped in the same cluster (VC_1 and VC_2 in Fig. 2).

These two clusters of GSB viruses showed different patterns based on shared gene content and host. In VC-2, three genomes assembled from the 2018 targeted metagenomes were clustered with a 34–59% shared gene content; of the three, one was associated with GSB-A, while the two others were associated with GSB-B, based on genome binning and co-detection in read mapping analyses. Based on their shared predicted gene content, the GSB viruses in this cluster appear to be recently diverged and co-diversifying each with a specific host population (Fig. 2). Conversely, in VC-1, one genome assembled from the 2018 targeted metagenomes was clustered, based on shared gene content, with one genome from the 2017 targeted metagenomes and one from the 2007 bulk metagenomes (Fig. 2). Comparisons of these three cluster members revealed two distinct regions of these viral genomes: a conserved region with predicted structural genes such as capsid and tail genes, and a variable region with mostly uncharacterized proteins. Hence, these three distinct temperate viral genomes likely represent different variants from the same population with different relative abundance through the years, consistent with an arms race dynamics [37]. This pattern of highly variable regions across years is an exception among GSB viruses: for the other three viruses identified in the targeted metagenomes and discussed further in this paper, identical or near-identical contigs (>95% ANI over >85% AF) could be identified in the 2005–2013 bulk metagenomes (Fig. S4).

Overall, our identified GSB viruses are distinct from all known viruses including one putative GSB virus from [27]), may represent six putative new genera, and appear to be genetically stable across decades except for one population that showed large changes in a hypervariable region. It is worth noting that, while both VC-1 and VC-2 have conserved and variable regions, they differ in that VC-1 has successive (but not co-occurring) variants within the cluster, while VC-2 is made up of co-occurring variants that appear to be relatively stable across time (Fig. S4). Further, for sequences in VC-3 and VC-4, contigs with identical or near-identical (>99%) on the viral regions could be assembled from 2005 to 2013 metagenomes (Fig. 2), suggesting these are also stable across time. This high degree of genetic stability across decades was unexpected, as most work on viral genome evolution has shown rapid genome change (of varying degrees) across short time scales, similar to what was seen in our VC-1 (which comprises virus CV-1–33) [37–39]. Genetic stability may be temporarily observed in the case of decaying prophages, i.e., prophages that can no longer enter the lytic cycle and thus become part of the host genome. However, in those instances, we would expect to observe visible signs of prophage degradation over the course of a decade such as gene loss and/or SNP accumulation, instead of the high stability observed here [40]. Hence, in the absence of such sign of prophage decay, we interpret these sequences as representing stable but active (or activable) prophages.

### Dynamics of CRISPR spacer acquisition and maintenance are highly variable between viruses

**Fig. 3:**
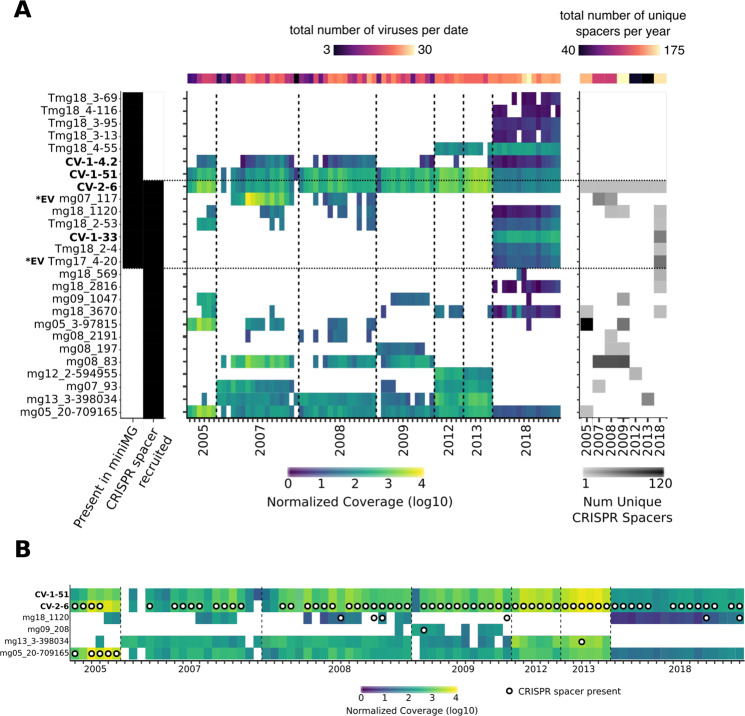
Long-term dynamics of GSB-associated viruses CRISPR spacers in TBL. **A** Shown are a subset of viral contigs identified through GSB CRISPR spacer matching, plus the identified GSB viral contigs through FACS flow sorting. A complete version of the heatmap is available as Supplementary Fig. S6. The four viral contigs discussed further in this manuscript are labeled in bold; variants of CV-1-33 are labeled as *EV in bold. *(left)* Black squares are used to signify which contigs were found in the targeted metagenomes (miniMG), and which contigs were recruited through CRISPR spacer matching. *(center)* Normalized coverage for each sample across all contigs; white space represents insufficient or no coverage for that contig/sample. *(right)* Shown are the number of unique CRISPR spacers detected in each year. **B** Shown are the same coverage values for GSB viral contigs, plus white with black circles that represent the presence of one or more CRISPR spacer(s) matching this virus in the corresponding samples.

Most (26) of the 45 viral OTUs detected in 2005–2013 based on CRISPR matches were not detected in 2018, either in targeted or bulk metagenomes (Figs. 3, S5, S6). A temporal comparison of viral contig coverage and the acquisition of their respective spacers revealed multiple instances of decline in virus coverage following spacer acquisition, suggesting successful defense by the host population against these infections (Figs. 3, S5, S6). However, we also observed a variety of other CRISPR-virus dynamics within our relatively simple host–virus system: viruses with no spacers (CV-1-51), viruses with unique/different spacers recovered each year (CV-2-6), viruses present for many years before spacers can be detected (mg13_3-398034), viruses that are found long after their spacers are no longer detectable (mg05_20-709165), spacers that are retained even after the virus seemingly disappears (mg18_1120), and viruses that disappear after the presence of spacers (mg088_83) (Figs. 3, S5, S6). Such diversity of CRISPR-virus dynamics in this one system suggests that spacers may have different levels of efficacy within a single host and different frequency within a host population, consistent with a distributed immunity model [41]. Eventually, these complex population-level interactions influenced by CRISPR spacer acquisition and viral infection dynamics (e.g., integration of prophages in the host genome) likely explain our observation that individual virus-host pairs can be associated with a broad range of CRISPR dynamics. This is also consistent with previous studies of virus-host model systems, which indicated that CRISPR-based immunity could be incomplete if based on only one of a few spacers, and that virus-driven host phenotype alteration may influence infection dynamics at the population level [42].

The fact that different GSB viruses are identified through targeted metagenomes and CRISPR spacer matching highlights how these two approaches are complementary, as they reflect different infection status (Fig. 3). While CRISPR-based recruitment identified many putative GSB viruses, most did not seem to represent ongoing, ecologically relevant infections but instead reflected past infections, as these spacers did not match to any recently recovered GSB virus (based on de novo assembly and read mapping, Figs. 3, S5). Conversely, viral sequences identified in targeted, flow-sorted metagenomes represent “in-cell” viruses, including integrated prophages not targeted by CRISPR-Cas systems, although this method may be less suitable for very rare (i.e., low rate of infected cells) or transient (i.e., short in-cell time) infections. Together these two approaches are thus highly complementary, identified dozens of viruses infecting GSB over a 13-year time period, and revealed these GSB populations were often simultaneously infected with >15 viruses at a single time point (Figs. 3, S5). Admittedly however, both approaches would not include most extracellular viruses, for which viral metagenomes generated from the same samples would be ideally required.

### Auxiliary metabolic gene identification in GSB viruses

Auxiliary metabolic genes (AMGs) are host genes found in bacterial viruses that can modulate host cell metabolism during infection, usually to increase the efficiency of viral replication. This phenomenon has been observed in many bacteria, including e.g., cyanobacteria, where some viral-encoded proteins are used during infection to maintain host cell photosynthesis capacity [43]. In GSB, comparative genomics previously revealed an extensive history of HGT, with the *sox* cluster for thiosulfate utilization as a well-known example [26]. It is thus possible that GSB viruses carry AMGs, in particular ones involved with sulfur metabolism.

Overall, we found no clear evidence for AMGs in any of the TBL GSB viral genomes. Specifically, none of the sequences identified as a putative GSB virus through targeted metagenome or CRISPR match included a predicted gene that could confidently be linked to a cellular metabolism. Since most (>60%) of putative GSB virus genes were not or poorly annotated however, it is possible that some GSB viruses do encode genuine AMGs, but further experimental characterization of these unknown genes will be required to identify these.

### Host–virus temporal dynamics differ across GSB populations in TBL

**Fig. 4:**
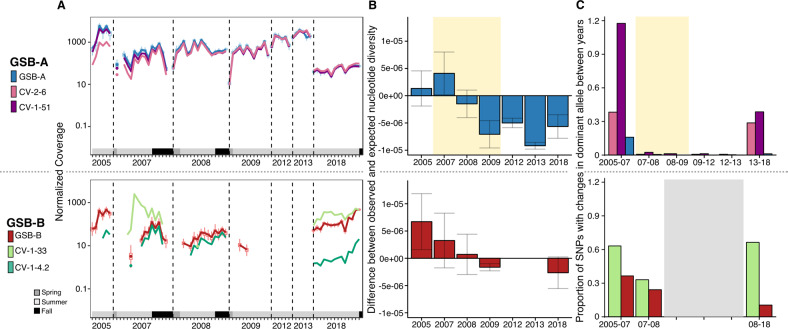
Relative abundance and population diversity of GSB hosts and viruses in Trout Bog Lake (2005–2018). **A** Normalized abundance (log-scale) of GSB-A and associated viruses (top) and of GSB-B and associated viruses (bottom); only samples with reads mapping to at least 25% of the genome are shown (Fig. S7), grey-scale boxes represent season of each sample. For 2018, coverage values are from standard bulk metagenomes, not from the targeted metagenomes. **B** Difference between observed and expected nucleotide diversity for GSB-A (top) and GSB-B (bottom). Because observed nucleotide diversity will be impacted by coverage depth (Supplementary Fig. S8), expected values of nucleotide diversity were calculated using a regression of observed nucleotide diversity and raw (not normalized) coverage across all samples. Bars show standard deviation, and yellow shaded region denotes time period during genome sweep. **C** Proportion of SNPs for which the dominant allele changes between years for GSB-A and associated viruses (top) and for GSB-B and associated viruses (bottom). For diversity and SNP assessments, samples were pooled per year, and years were excluded from analysis if there was less than a 10× coverage for each SNP; yellow shaded region denotes time period during genome sweep; gray region represents years with low coverage, thus not enough data for accurate SNP analysis. Although one targeted metagenome was generated in 2017, no bulk metagenome was available for this year, and it is thus not included in these analyses.

Mapping reads from individual bulk metagenomes to the new GSB genomes and GSB viruses assembled from targeted metagenomes enabled a more detailed investigation of the temporal dynamics of GSB and their viruses in TBL. These data provide a unique look at virus-host dynamics, as these data cover more than 10 years, and represent one of the longest running datasets currently available to investigate viral–host dynamics in natural systems. Based on read mapping, GSB-A was present in all years dating back to 2005, and was, until 2018, the dominant host population (Figs. 4A, S7). Its associated viruses (CV-2-6 and CV-1-51, both integrated prophages) appear to be present at a low relative abundance in 2005, and based on coverage, became pervasive in 2007, seemingly infecting every member of the host population from this date on. While both host and viruses decreased in abundance in 2018 relative to previous years, they were still abundant as judged by coverage (>40×; ~32% of GSB reads in 2018 were GSB-A) and showed the expected increase in summer compared to spring samples corresponding to the GSB bloom. While this long-term virus-host stability was unexpected, as viruses are often thought of as fast-evolving, recent work done in marine systems also showed long-term stability in viral communities, resulting in long-term virus-host co-existence [38]. The mechanisms for this stability are likely different however, as those coastal marine systems seem to rely on perpetually changing minor variants (Red Queen Hypothesis [44]), while no evidence of such an arms race was observed in TBL GSB-A viruses: the coverage of both host and viruses were nearly identical, no change in viral genome content was observed, and no CRISPR spacers targeting these GSB-A viruses were detected.

In contrast, GSB-B was overall less abundant, with reliable coverage (>10×) only achieved in 2005, 2018, and some samples in 2007. While it was not the dominant GSB population for most of the years covered by our data, it was clearly more abundant than GSB-A in 2018 (Figs. 4A, S5). Similar to GSB-A, GSB-B is also associated with an integrated prophage (CV-1–4.2). However, unlike the prophages that infect GSB-A, CV-1-4.2 was substantially less covered than its host GSB-B at most time points (15 times less on average for dates when the virus was detected), likely infecting only a subset of the population (Fig. 4A). The other GSB-B virus (CV-1-33) displayed a strikingly different virus-host dynamic than all the other GSB viruses we observed, with large peaks in abundance compared to its host in 2007 and 2018. We interpret these abundance peaks where the coverage of the virus vastly exceeds the coverage of the host as reflecting the presence of multiple copies of the viral genome in individual host cells and/or the presence of encapsidated genomes in the sample stemming from e.g., virus particles stuck onto cell clumps, which would both be signs of ongoing viral replication and lytic infections. It is notable that CV-1-33 is also the only virus that shows high mutation/recombination rates with a clear hypervariable region (see above and Fig. 2), which would be consistent with a lytic virus engaged in an “arms race” with its host. These data also confirm that virus-host association based on relative abundance correlation could be efficient for prophages but may misinterpret the dynamics of lytic viruses (Fig. 4), as predicted by theoretical models [45].

At the community level, a mix of persistent viruses (repeatedly detected across seasons and years) and transient viruses (detected in only one or a few time points) have been reported in marine systems [46–48] and in lakes [1, 49]; however, the ecological and evolutionary drivers of virus persistence are still unclear. Here, our data show that the viral community associated with individual host populations can include both persistent and nonpersistent viruses. In addition, while persistent viruses tend to be associated with lysogenic infections, CV-1-33 was notable as a persistent virus predicted as lytic, displaying molecular arms race dynamics, and prevalent enough to be detected in targeted metagenomes. This suggests that the two strategies may enable virus persistence: successful arms race leading to multiple virus variants appearing and disappearing through the years, and long-term stable co-existence of temperate viruses with their hosts. The fact that both strategies were detected in closely related GSB species in the same environment (TBL) with similar host growth dynamics and nutrient availability suggests that other factors may drive the success or failure of these different virus strategies.

### Genome-wide sweep affected one GSB population and its associated viral populations

As previously reported in ref. [31], GSB-A went through a genome-wide sweep (i.e., an event where a genotype within the population increases in frequency to reach nearly 100% and becomes fixed, reducing population diversity to nearly zero) in 2007, leading to a stark reduction in population diversity (Fig. 4B). The same pattern could be observed here for the associated viruses, which both underwent a clear genome-wide sweep (Fig. S8). In 2018, we are able to see some higher level of population diversity compared to 2013 in terms of single nucleotide variants, especially in the viruses (Figs. 4B, S9), suggestive of an ongoing slow diversification process. Viral infections could have been a possible cause of the genome sweep seen in GSB-A, because we can see an increase in prophage abundance concomitant with a decrease in GSB-A population diversity (Fig. S9). On the other hand, the genome sweep may have provided the appropriate conditions for the GSB-A viruses to attain high levels of abundance if these two viruses happen to infect the strain of GSB-A that became dominant after the sweep, reminiscent of the “piggyback-the-winner” hypothesis [9]. For GSB-B, we were not able to reliably ascertain population diversity in 2012, 2013, and most of 2009 because of a low coverage depth, however neither GSB-B or its associated viruses seemed to undergo a genome sweep comparable to GSB-A (Fig. 4B).

In addition to estimating microdiversity within GSB hosts and their associated viral populations each year, we also looked at the turnover between populations each year by calculating the proportion of SNPs for which the dominant allele changed between years. This was calculated similar to our standard SNP calculations, but instead of looking at overall SNPs density across all samples, pairwise comparison of years was computed with samples per year pooled. By looking at turnover in the GSB populations, we can estimate how similar (or dissimilar) a given population is compared to the population in the year before or after. In GSB-A, we see a similar pattern as the within-sample nucleotide diversity, with higher SNP turnover between 2005 and 2007, and between 2013 and 2018 (Fig. 4C), suggesting that the GSB populations within these time periods were less similar than between 2007 and 2013. Between 2007 and 2013, the same clonal GSB-A population dominated every year, as little-to-no SNP changes between or within years were observed. For the GSB-A associated viruses, we see greater turnover between years than we saw in the host between 2005–2007 and 2013–2018, however, between 2007 and 2013, turnover in the viruses is at a similar near-zero level as seen in the host genome (Fig. 4C). Notably, the vast majority of the SNPs observed (98%) corresponded to synonymous mutations, further suggesting these viruses are not “decaying prophages”, i.e., these are not inactive viruses progressively degrading in the host genome, as already suggested based on gene content (see above). In GSB-B, we were only able to calculate turnover between 2005–2007, 2007–2008, and 2007–2018 due to a low coverage in the other years, but GSB-B population overall showed a higher turnover compared to GSB-A (Fig. 4C). Notably, SNP turnover in CV-1-33 were not restricted to the “variable” region but also included SNPs in conserved genes. CV-1-33 is thus a uniquely dynamic genome, both at the gene content level (gene replacement from year-to-year) and SNP level (allele turnover in conserved genes) (Fig. 4C).

### Contrasting host population-level diversity may drive viral infection dynamics

Although GSB-A and GSB-B grow under similar ecological conditions and harbor similar genomes (Table S2), these two hosts appear to experience different types of viral infections. GSB-A is associated with persistent prophages with high prevalence rate (infecting nearly 100% of the host population), while GSB-B is associated with two different viral types: a rare prophage infecting a subset of the population, and a (likely) lytic virus with a rapidly evolving genome. Because GSB-A went through a genome-wide sweep, which resulted in decreased population diversity, we hypothesized that host population diversity could explain this difference in infection types, especially as it could impact the resistance potential of each host population. For clonal or nearly clonal host populations such as GSB-A in TBL, where the dominant strain is infected by a virus, the probability of a resistant strain appearing would be initially low because there are few uninfected hosts with potential for resistance in the population. From the virus perspective, the most successful strategy once most of the host population has been infected would thus be a long latent/lysogenic cycle that sustain a stable co-existence of virus and host. In contrast, a primarily lytic virus would likely remove a substantial portion of the host population, and a resistant host strain would eventually arise despite the host population being originally clonal [50].

In contrast, for populations with higher levels of strain diversity such as GSB-B, there is likely an existing pool of strains with potential for resistance. For this population, instead of stable prophages, we observe either a “rare” prophage, or a rapidly changing lytic virus that would be consistent with ongoing kill-the-winner/Red Queen type dynamics. Hence, in higher-diversity host populations, the most successful strategy for viruses could be lytic or short lysogenic infection cycles, leading to more replication events and a more diverse viral population. Temperate viruses infecting these higher-diversity host populations, while less successful (in terms of relative abundance and infection rate), could still be stably associated with a subset of the host population as observed here in the GSB-B virus, CV-1-4.2.

### Theoretical model suggests low host population diversity favor lysogenic infections

To examine the potential for these scenarios to occur under different conditions, we adapted an existing theoretical model [6] to consider the relationship between lysogeny, induction rate, and host resistance/susceptibility. As a first step, we were interested in identifying conditions that would allow for maximum lysogeny (i.e., majority of the host population are lysogenized), versus those favoring lytic activity (see Methods). While our model does not include a direct measurement for diversity, we used the initial abundance of lysogenized host cells as a simple proxy. Indeed, the genome-wide sweep in GSB-A was associated with an increase in the percentage of lysogenized cells, and while we can’t distinguish if one directly caused the other, a population bottleneck event could lead to a higher frequency of lysogenized cells (Fig. S9). Meanwhile, our model allows for hosts to transition from susceptible to resistant, but not from lysogenized to resistant, so resistance would have a greater chance to arise in a diverse population with a lower initial proportion of lysogenized hosts because there is a greater proportion of hosts that could become resistant.

**Fig. 5:**
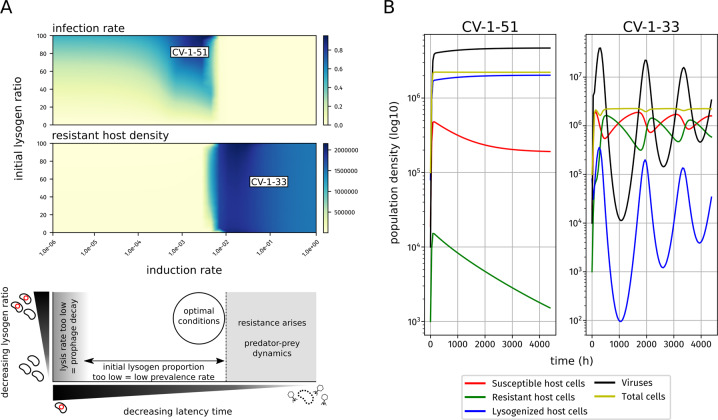
Theoretical modeling of virus-host dynamics with varying host population diversity. **A** Relationship between induction/lysis rate, initial ratio of lysogenized host cells, and either final infection rate (top) or resistant host density (center). Combintion of induction rate and initial ratio of lysogenized host similar to CV-1-51 and CV-1-33 GSB viruses are labeled on the plot, and used to generate the vignettes in the right panel; (bottom) shows a summary schematic of the above plots and the corresponding virus-host dynamics; the “optimal” conditions maximize the amount of hosts a given virus can infect. **B** vignettes for CV-1-51-like (left) and CV-1-33-like (right) dynamics; population density for each respective group (see legend and Methods) are plotted on a log10-scale against time (*x*-axis, 9 months total).

Across the full landscape of induction rate and initial lysogen ratio, our model broadly recapitulates known virus-host dynamics. Most scenarios leading to a high rate of lysogens (dark blue region in top panel of Fig. 5A), i.e., a complete or near-complete infection of the host population at the end of the simulation, started from high (40% or higher) initial rates of lysogeny (Fig. 5A, *y*-axis). However, this final lysogen rate was also dependent on induction rate (*x*-axis on Fig. 5A). If the induction rate was too low, then lysogenic infection rate decreases due to prophage decay. On the other end, higher initial lysogen ratio are associated with low decreased lysogen ratio and increased resistant host density (Fig. 5A). This suggests that there is a tipping point in this model, where a sufficiently high induction rate will allow the virus-host dynamics to enter into predator-prey/Red Queen dynamics leading to lower final lysogen ratios.

We can further examine these virus-host dynamics by modeling individual vignettes with specific conditions that attempt to mimic those from two of our GSB viruses, and deduce the possible scenarios that gave rise to the virus-host dynamics observed in TBL. We first verified that our model recovered expected predator-prey dynamics for lytic viruses by testing a case similar to CV-1-33, a virus with low latency (i.e., mostly lytic) and ~50% infection rate (Fig. 5B). Here we see expected cyclical dynamics for viruses, resistant hosts, and susceptible hosts, consistent with the relative abundance patterns observed for CV-1-33 (Fig. 5B). Next, we used this model to investigate the conditions that may enable the long-term maintenance of a stable prophage like CV-1-51. CV-1-51 infects most (if not all) of the GSB-A population, and has remained stable for over 10 years. In this scenario, we modeled a low induction rate, and started the time course with an initial lysogen/susceptible host ratio of 30%. For the 9 months over which the model was run, we find a slow but steady increase in lysogenized host cells, and no significant rise of resistant host cells (Fig. 5B). Similar results were obtained for the same induction rate and a higher ratio of lysogen hosts, as could have occurred following the genome sweep observed in GSB-A (Fig. 5A). Hence, with low levels of induction, and especially following a genome-wide sweep which would have increased its prevalence, CV-1-51 can be maintained within a near-clonal host population without viral genome decay nor triggering a rise in host resistance. While more sophisticated mechanisms for this type of stability may exist, such as density dependence induction rate [51], we did not find evidence of those mechanisms occurring here, and our model suggests that this type of stability is possible even without those.

## Conclusion

In this study, we characterize viruses infecting GSB in a model freshwater lake. By leveraging the unique autofluorescence of GSB cells, we sorted GSB cells using FACS, and sequenced these “targeted metagenomes” to identify GSB-specific viruses. These targeted metagenomes allowed us to observe cell-associated viruses, and ecologically relevant ongoing infections. Overall, this newly developed method recovered novel viruses with a host context at high (population-level) resolution. These new host-contextualized viruses in turn enabled a re-analysis of time series bulk metagenomes to investigate virus-host interactions across seasons and years in a model freshwater lake. For one virus persistent through multiple years (CV-1-33 infecting GSB-B), we observed temporal dynamics and microdiversity patterns consistent with a “kill-the-winner” dynamic. However, for a closely related host population, viruses persisting throughout multiple years seem to be instead temperate and consistent with a “piggyback-the-winner” dynamics (CV-1-51 infecting GSB-A). A theoretical model of these infections suggest that host population diversity may be a critical factor driving these virus-host interactions by influencing the potential for resistance in each host population. “Piggyback-the-winner” strategies would be more successful when prophages with low induction rates are able to spread through low-diversity host populations and infect all members of the dominant strain, while “kill-the-winner” dynamics would more frequently occur in high-diversity host populations which include a subset of resistant cells. Consequently, the optimal latency time decision of viruses would in turn depend partly on the host population diversity, with long-latency prophages favored in low-diversity host populations. This would be consistent with the link between lysogeny and host life-history traits, as lysogeny-associated traits such as “pathogenicity” or “boom-and-bust” life cycles are also associated with population bottlenecks, and could also explain the imperfect linkage between lysis-lysogeny rate and broader ecological parameters such as host abundance, nutrient availability, and growth rate. While this study focuses on one environment, two specific host populations, and their “in-cell” viruses, it would be of interest to verify if the general patterns observed also hold true for other GSB populations in other lakes, and for other bacterial populations with varying growth cycles and dynamics.

## Materials and methods

### DNA sampling and sequencing

TBL is located in Vilas County, Wisconsin, USA, and is surrounded by a *Sphagnum* mat that supplies large amounts of terrestrially derived organic matter to the lake, leading to darkly stained water and greatly attenuated light penetration. The lake mixes twice per year (spring and fall) but thermally stratifies strongly in the summer. It has a maximum depth of 7 m, surface area of ~11,000m^2^, and a mean pH of 5.1 [52]. Dissolved oxygen is typically below detection in the hypolimnion (lower layer of water in a stratified lake) between ice thaw (early May) and fall mix (mid-November) ([52], https://lter.limnology.wisc.edu/researchsite/trout-bog); these yearly temperature changes along with other major biogeochemical parameters can be seen in Fig. S2.

Two different types of samples were collected during June-October 2018: (1) depth-integrated water samples were collected onto filters (see below) from the hypolimnion layer (as defined by the temperature gradient) at 14 different time points, and DNA was purified from these filters using the FastDNA Spin kit for Soil (MP Biomedicals, Santa Ana, CA), with minor modifications to the kit protocol, as described previously [52]; (2) depth-discrete water samples were collected at specific depths throughout the water column at 14 different time points (Table S4), preserved in glyTE (1 ml of sample for 0.1 ml Gly-TE buffer), and were counted and sorted using flow cytometry (see below). Details about the sample types and depths can be found in Fig. S1 and Supplementary Table S4. Both types of samples were filtered onto a 0.2-μm pore-sized polyethersulfone filters (Supor 200, Pall, Port Washington, NY), without pre-filtration to ensure a consistent sampling protocol through the entire time series, prior to storage at −80 °C. Specifically, depth-integrated samples were kept cold at 4 °C for ~1 h, and frozen at −20 °C after filtration (due to field sampling limitations). These samples were then transported to Madison, WI, USA on dry ice and transferred to −80 °C as soon as possible, and always within 2 months. Depth-discrete samples used for cell sorting were instead transferred immediately to dry ice and shipped overnight to JGI for processing (see below).

For the depth-integrated samples (i.e., “bulk” metagenome), one library from each sample was prepared following standard KAPA kit protocol. 10 ng of Genomic DNA was sheared to 300 bp using the Covaris LE220 and size selected with SPRI using TotalPure NGS beads (AMPure XP, Bechman). The fragments were treated with end-repair, A-tailing, and ligation of Illumina compatible adapters (IDT, Inc) using the KAPA Standard Library Creation kit (KAPA Biosystems) and five cycles of PCR was used to enrich for the final library.

For the depth-discrete samples (i.e., “targeted” metagenomes), Nextera XT v2 (Illumina) sequencing libraries were generated from 5,000 sorted cells following multiple displacement amplification (MDA) [53]. Five libraries of sorted GSB, i.e., pigmented cells that were stained by SYBR Green (Invitrogen) (488 nm excitation; 530 nm emission) and emitted autofluorescence >750 nm after excitation with 642 nm laser (Fig. S10), and two additional libraries of sorted nonpigmented cells were sequenced for each sample. Sorted cells were amended with glycerol (6% final concentration) to protect them from unintended lysis during the freeze/thaw process, and stored at −80 °C while awaiting MDA. Prior to amplification, cells were pelleted by centrifugation at 6350 × *g* for 1 h at 10 °C, inverted and centrifuged at 7 × *g* for 5 s to remove supernatant, and amplified by MDA at 45 °C for 16 h using EquiPhi29 DNA polymerase (ThermoFisher Scientific) following the protocol described in ref. [54]. Paired-end sequences of 2 × 150 bp were generated for all libraries on the NovaSeq platform (Illumina). Metagenomic sequence reads are publicly available on the JGI IMG portal and NCBI SRA (Table S5).

### GSB cell flow cytometry cell sorting

Benchmarking for this protocol was performed on cultures of *C. tepidum*. Briefly, the influx cell sorter (BD Biosciences) was prepared following the protocol for single-cell genomics outlined in [55]. However, instead of sorting individual cells, five pools of 5,000 sorted GSB cells and two pools of nonpigmented cells were sorted into reaction wells from each depth-discrete sample. GSB were identified as pigmented cells that were stained by SYBR Green (Invitrogen) (488 nm excitation; 530 nm emission) and emitted autofluorescence >750 nm after excitation with 642 nm laser [56, 57], whereas the nonpigmented cells were identified as those stained by SYBR Green and lacking autofluorescence (Fig. S10). Sorted cells were amended with glycerol (6% final concentration) and stored at −80 °C while awaiting MDA.

### Data processing and genome binning

For depth-discrete water samples (hereafter “targeted metagenome”), each dataset was subsampled to 60 million total reads per sample using BBMap reformat function [58]. For each sampling date, reads from the five replicates were pooled and coassembled using SPAdes (v3.10.1, --phred-offset 33 -t 16 -m 120 --sc --careful -k 25,55,95–12 [59]). For each coassembly, both positive and negative cell fractions were read-mapped to the contigs using Bowtie2 with default parameters [60]. To remove any cross-talk contamination during sequencing, only contigs that had at least 10× higher coverage in the positive fraction (compared to the negative fraction) were kept for downstream analysis. After filtering, contigs for each coassembly were binned based on CONCOCT clustering using Anvi’o v5.5 [61] and bin quality was determined using CheckM [62]. Once binning was complete for each coassembly, all bins were pooled and dereplicated using dRep with default parameters [63]. The dereplicated genome set was used as the representative genomes across all samples in 2018. Both depth-integrated and depth-discrete metagenomes were read-mapped to these representative genomes using Bowtie2, and coverage data was extracted using Anvi’o v5.5 [61].

### GSB genome bin annotation

TBL GSB genome bins were annotated using Prokka (v1.13.4, --metagenome, [64]). Genes for pigment biosynthesis (bchU, cruB, bciD) were identified in the GSB metagenomes both through gene annotation (using Prokka, see above) and blastp matching to known GSB pigments genes [34, 65–67]. CRISPR arrays were predicted using CRT1.2 [68], with default parameters. Predicted arrays were manually inspected to make sure that repeats were all exactly of the same length, identify arrays with identical repeats within and between bins, and check the presence of nearby Cas genes when enough of the neighboring region was assembled.

### Virus identification and characterization

To identify potential viral contigs, contigs from all 14 2018 co-assemblies of sorted cells and all bulk metagenome assemblies were analyzed by VirSorter 1.0.5 [69]. Briefly, VirSorter looks for hallmark viral genes (e.g., tail proteins, terminase, capsid protein) and enrichment of viral-like genes in the contigs provided, and ranks the contigs based on these characteristics along with matches to known viral genomes. Sequences identified by VirSorter in categories 1, 2, 4, and 5, were manually curated (by looking at genome structure and content for known viral genes), and genuine viral contigs from each coassembly were pooled and dereplicated (using dRep [63],) to produce a representative set of potential GSB viruses. Genome figures for viral genomes were generated using EasyFig (v2.2.2, [70]), with the annotations produced from VirSorter. Viral contigs covered at >5× in at least 50% of replicates of a set of GSB-targeted metagenomes (i.e., one sampling date) were considered as GSB viruses.

To identify contigs from putative GSB viruses using matched CRISPR spacers, all metagenomes were first processed with the Crass assembler (v0.3.12) [71], to broadly assemble CRISPR arrays. Then, the repeats predicted for the Crass-assembled arrays were compared to the repeats identified in CRISPR arrays predicted in both GSB genome bins (see above), and Crass-assembled arrays with repeats identical to one of the GSB arrays were considered as “GSB-encoded” arrays. Spacers associated with these GSB-encoded arrays (Table S6) were gathered and compared to all viral contigs identified across all metagenomes (see above) using blastn with options optimized for short sequences (“-dust no -word_size 7”). Predicted viral contigs showing at least one match to a GSB-associated spacer (0 or 1 mismatch across the whole spacer length) were selected as “candidate GSB viruses”.

### Taxonomic classification of predicted GSB viruses

For taxonomic affiliation, all viral contigs identified (including the ones not associated with GSB) with a length ≥10 kb were pooled with reference genomes from NCBI RefSeq (v93, filtered to include only genomes from bacterial and archaeal viruses), along with viral sequences from the IMG/VR database with a predicted GSB host [5], prophages predicted in GSB genomes from the IMG database [72], sequences predicted with VirSorter (categories 1/2/4/5), and viral sequences ≥10 kb identified in metagenomes from Lake Mendota (predicted with VirSorter as for TBL metagenomes, see above). The additional datasets beyond RefSeq were included to try to ensure a large representation of sequences from freshwater viruses and/or putatively associated with GSB. This set of viral genomes and contigs was first dereplicated (95% ANI, 85% AF) using MUMMER 4.0.0b2 [73]. The resulting dataset was then used as input to build a genome network based on shared gene content using vContact 2 ([74], using diamond [75] for all-vs-all protein comparison, MCL for protein clustering [76], and ClusterOne for genome clustering [77]. The resulting network was visualized using Cytoscape [78].

### Time series dynamics

Depth-integrated samples (referred to as “bulk metagenomes” here) were previously taken from TBL in 2005, 2007–2009, and 2012–2013 [31]. In order to conduct a time series analysis of GSB and their viruses, reads from those samples were mapped to both the representative GSB genome set and the representative GSB virus set (see above) using Bowtie2. Coverage data was extracted using Anvi’o v5.5 [61].

### SNP identification and analysis

SNPs were identified using Anvi’o v5.5 [61]. Only SNPs with at least 10× coverage in each sample (Fig. 4A) or year (Fig. 4B, C) were retained for analysis. Allele frequencies were rarified to a depth of 10, also for each sample/year, and SNPs were filtered again to exclude those SNPs whose dominant allele frequencies were now 1. Nucleotide diversity (Pi) was calculated for each SNP, values were summed (per sample), and divided by genome size. SNP turnover was calculated in a similar way by comparing combined allele frequency of individual years to each other.

### Virus-host model

The virus-host model used here was based on [6], with *p* (prophage decay rate, h^−1^) and *cost* (growth cost) as additional parameters, and includes the following nonlinear ordinary differential equations:

$$\begin{array}{l}{\mathrm{Susceptible}} = S = \overbrace {bS\left( {1 - \frac{N}{K}} \right)}^{{\mathrm{growth}}} - \overbrace {\varphi SV}^{{{\mathrm{infected}} \atop {({\mathrm{to}}\;{\mathrm{L}})}}} - \overbrace {dS}^{{\mathrm{death}}} - \overbrace {\mu S}^{{{\mathrm{mutated}} \atop {({\mathrm{to}}\;{\mathrm{R}})}}} + \overbrace {\mu R}^{{{\mathrm{mutated}} \atop {({\mathrm{from}}\;{\mathrm{R}})}}} + \overbrace {pL}^{{{\mathrm{prophage}} \atop {\mathrm{decay}}}}\\ {\mathrm{Resistant}} = R =\overbrace {rbR\left( {1 - \frac{N}{K}} \right)}^{{\mathrm{growth}}({\mathrm{with}}\;{\mathrm{cost}})} - \overbrace {dR}^{{\mathrm{death}}} - \overbrace {\mu R}^{{{\mathrm{mutated}} \atop {({\mathrm{to}}\;{\mathrm{S}})}}} + \overbrace {\mu S}^{{{\mathrm{mutated}} \atop {({\mathrm{from}}\;{\mathrm{S}})}}}\\ {\mathrm{Lysogens}} = L = \overbrace {cost * bL\left( {1 - \frac{N}{K}} \right)}^{{\mathrm{growth}}({\mathrm{with}}\;{\mathrm{cost}})} + \overbrace {\varphi SV}^{{{\mathrm{infected}} \atop {({\mathrm{from}}\;{\mathrm{S}},{\mathrm{V}})}}} - \overbrace {nL}^{{\mathrm{lysis}}} - \overbrace {dL}^{{\mathrm{death}}} - \overbrace {pL}^{{{\mathrm{prophage}} \atop {\mathrm{decay}}}}\\ {\mathrm{Virus}}\;{\mathrm{particles}} = V \,=\, \overbrace {\beta nL}^{{\mathrm{new}}\;{\mathrm{lysis}}\;{\mathrm{events}}} \,-\, \overbrace {\varphi SV}^{{{\mathrm{infected}} \atop {({\mathrm{to}}\;{\mathrm{L}})}}} - \overbrace {\varphi LV}^{{{\mathrm{infected}} \atop {({\mathrm{already}}\;{\mathrm{L}})}}} - \overbrace {mV}^{{\mathrm{virion}}\;{\mathrm{decay}}}\end{array}$$

S, R, L, and V denote the densities of susceptible cells, resistant cells, lysogenic cells, and virus particles per ml, respectively. N denotes the total density of cells, i.e., S + R + L. The parameters similar to [6] include *b* (maximal cellular growth rate, h^−1^), *K* (carrying capacity, ml^−1^), *φ* (adsorption rate, ml/h), *d* (cellular death rate, h^−1^), *β* (burst size, number of virus particles), *m* (virion decay rate, h^−1^), and *n* (induction rate, h^−1^). Additional parameters are *μR* (mutation rate from susceptible to resistant, h^−1^), *μS* (mutation rate from resistant to susceptible, h^−1^), *p* (prophage decay rate, h^−1^), *r* (effect of resistance on cell growth), and *cost* (effect of lysogeny on cell growth). Cell death (unrelated to phage infection) was kept identical across all cell types. The adsorption of virus particles to susceptible and lysogenic cells was also kept identical, with the latter being an evolutionary dead-end (i.e., no new lysogenic cells or virus particles are produced from these events), and the adsorption to resistant cell was considered as null (i.e., the resistance was assumed to arise from an absence of adsorption of the virions to the resistant host cell).

The two parameters varying between computations were n (induction rate) from 1 × 10^−6^ to 1 and the initial proportion of lysogenic cell from 0 to 100%. The initial number of cells was set at 1 × 104 ml^−1^ based on the flow cytometry counts of GSB for the first samples in our 2018 time series, while the maximum carrying capacity *K* was set at 3 × 10^6^ based on the maximum count of GSB throughout the 2018 time series (see above). The initial number of viral particles was set at 1 × 104 ml^−1^ (i.e., a virus to host ratio of 1). Additional simulations were run with an initial number of viral particles of 1 × 105 ml^−1^ and 1 × 106 ml^−1^, and the same dynamics and patterns were observed as the ones presented in Fig. 5. Growth rate and washout rate were set at 0.02 and 0.005, respectively, based on previous measurements of growth for Chlorobiales strains in the laboratory [79]. These growth and washout rates enabled the formation of GSB blooms, i.e., a cell concentration increase from 1 × 104 to 3 × 106, in ~6 weeks, as observed in TBL. This growth rate, corresponding to a doubling time of ~35 h, is relatively low compared to most other bacteria, but is consistent with growth rates of known Chlorobiales strains both in laboratory cultivation and in the environment [79].

No measurement could be made or obtained from the literature for the other parameters, which were thus set to standard values as follows: Burst size, adsorption rate, and viral decay were set based on [6], i.e., *β* = 50, φ = 6.7 × 10 − 10 ml h^−1^, and *m* = 1/48 h^−1^, which correspond to standard phage infection dynamics. This virion decay rate of ~0.015 is within the range of values previously observed in freshwater systems, although decay rate has not been measured for GSB phages specifically [80, 81]. Prophage decay rate *p* was set at 1 × 10^−5^ h −1 corresponding to ~ 0.05% of prophage loss per generation at the maximum growth rate of 0.02 h^−1^. Mutation rate from susceptible to resistant (μR) and from resistant to susceptible (μS) was arbitrarily set at 1 × 10^−6^ to ensure a constant pool of both susceptible and resistant host cells in the system. Finally, the adjusting parameter for lysogenic growth rate was set at 0.99 to reflect the added energetic cost of the integrated prophage, and the adjusting parameter for resistant cells growth rate was set at 0.90 to allow for the establishment of typical prey-predator dynamics for high induction rate (i.e., lytic phages).

Simulations were run separately for a combination of lysis rate (30 values from 1 × 10^−6^ to 1 evenly distributed on a base-10 log-scale) and initial proportion of lysogenic cells (0–100% by increment of 5%) for 6570 h, i.e., ~9 months, corresponding to a full seasonal bloom of GSB as observed in our time-series. Since we do not have any data about host cell and virus:host dynamics during the time between blooms, i.e., late fall, winter, and early spring, for which we do not have samples, we opted to not run the model across multiple years. Infection rate and resistant host density data were compiled from the second half of each simulation (i.e., the last 3285 h), i.e., after the initial growth of the GSB population and once the bloom was established. For Fig. 5, the minimum value of each parameter (infection rate and resistant host density) across the second half of each simulation is used in the heatmap, to avoid biases linked to short peaks of infection or resistance.

## Supplementary information

Supplementary Figures

Supplementary Table 1

Supplementary Table 2

Supplementary Table 3

Supplementary Table 4

Supplementary Table 5

Supplementary Table 6
