# Supplementary material for: Host population diversity as a driver of viral infection cycle in wild populations of green sulfur bacteria with long standing virus-host interactions
Source: ISME J. 2021 Jan 15;15(6):1569–84. doi: 10.1038/s41396-020-00870-1 (PMC8163819; doi:10.1038/s41396-020-00870-1)
Supplement: Supplementary file 1 — Supplementary Figures [file 41396_2020_870_MOESM1_ESM.pdf]

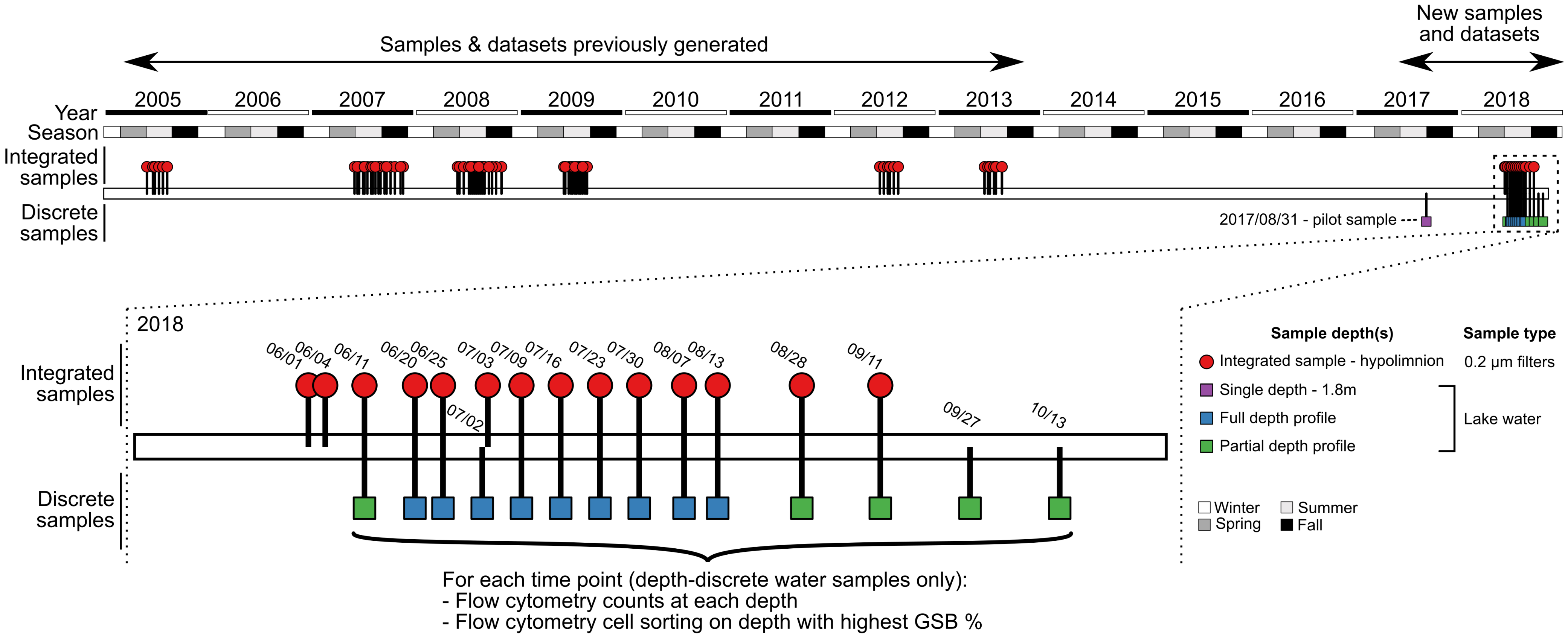

Figure S1. Overview of the sampling strategy. Two types of datasets were used in this study. Bulk metagenomes were generated from integrated samples of Trout Bog Lake hypolimnion (red circles) for which cells were collected on 0.2 µm filters. Bulk metagenomes from 2005 to 2013 were previously generated and published (Bendall, et al. 2016). Bulk metagenomes from 2018 were sampled and generated for this study, using a similar approach as the 2005-2013 samples to generate comparable data. Water samples were collected specifically for this study from one time point in 2017 (pilot sample, purple square) and 13 samples in 2018 (blue and green squares). In 2018, two types of depth profiles were sampled. A full depth profile included samples from 0.5m to 5m by increment of 0.5m, along with samples at 6m and 7m (blue). Partial depth profiles included samples from 1m to 4m by increment if 1m (green). Depth-discrete water samples were used for (i) flow cytometry count of total cell number and estimated number of GSB cells (based on pigmented cells detection, see Methods), and (ii) flow cytometry cell sorting of GSB cells from the single depth with the highest GSB percentage, for each time point.

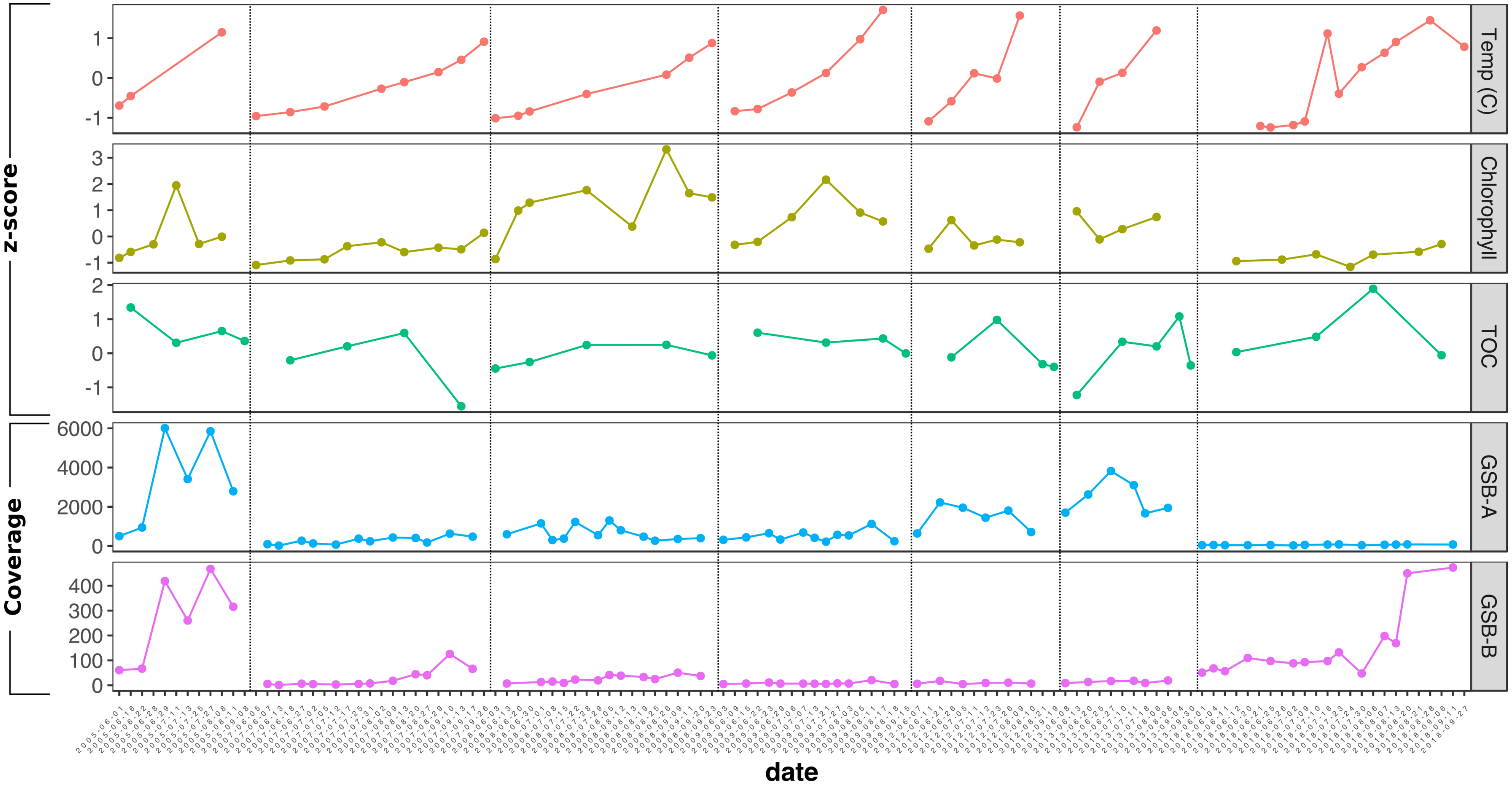

Figure S2. (top) Z-scores for environmental data at Trout Bog Lake (chlorophyll, lake temperature, and total organic carbon (TOC)) shown from 2005-2018. (bottom) Normalized coverage values for GSB-A and GSB-B.

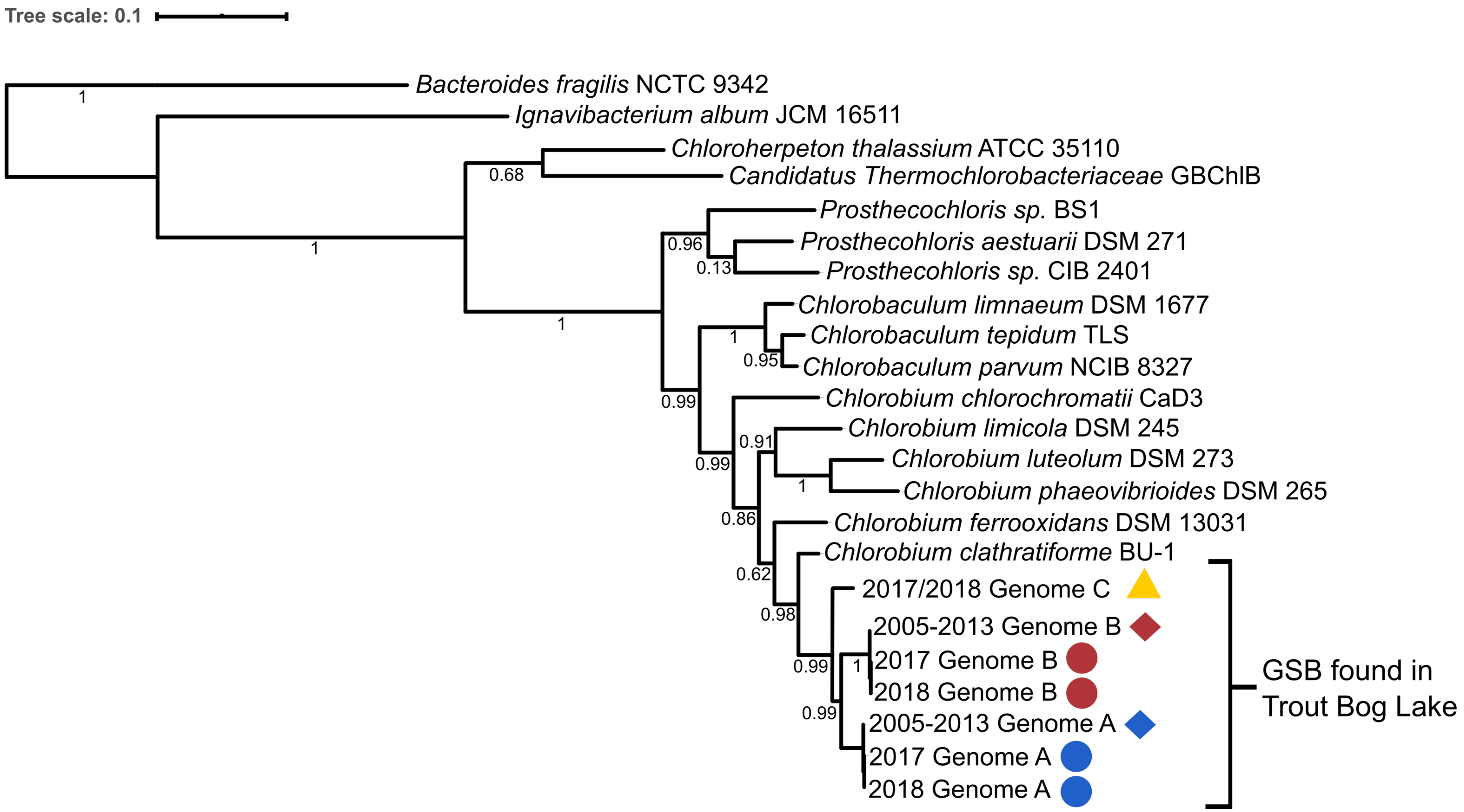

Figure S3. Phylogenetic tree based on DNA sequence of rpoB (beta subunit of RNA polymerase) genes of GSB from Trout Bog Lake and GSB isolate genomes; circles represent new genome bins from our targeted metagenomics, and diamonds represent previously published genome bins (Bendall et al., 2016); colors represent Genome C (yellow), Genome B (red), and Genome A (blue).

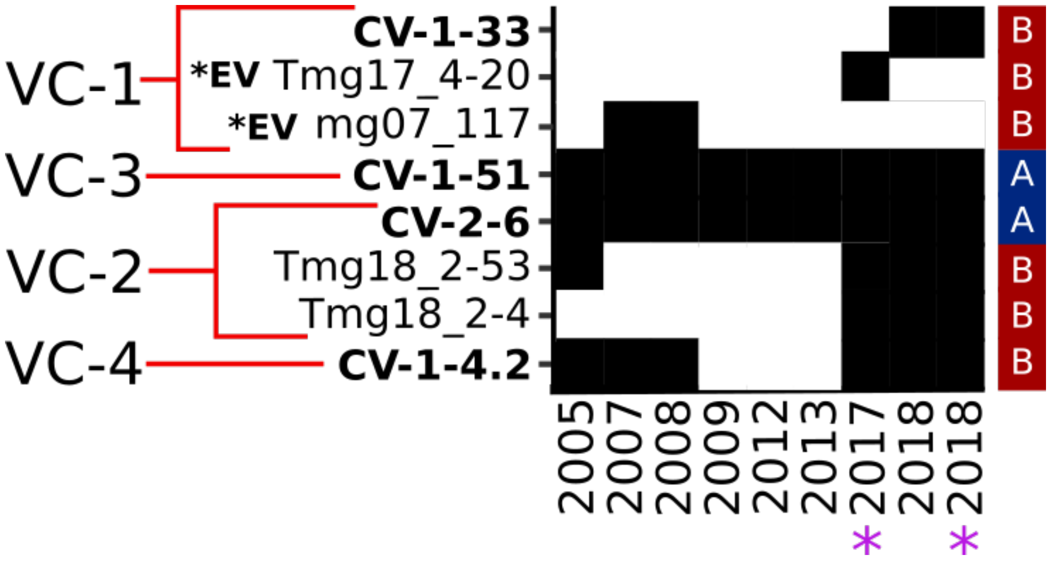

Figure S4. Shown are presence/absence values for viral clusters shown in red in Figure 2. The four viral contigs discussed in this paper are labeled in bold; variants of CV-1-33 are labeled as *EV in bold. Shown is presence/absence for each year across all contigs; starred years represent the targeted metagenomes, where at least half of all replicates must contain the contig to be counted as present that year. Hosts are labeled on the right.

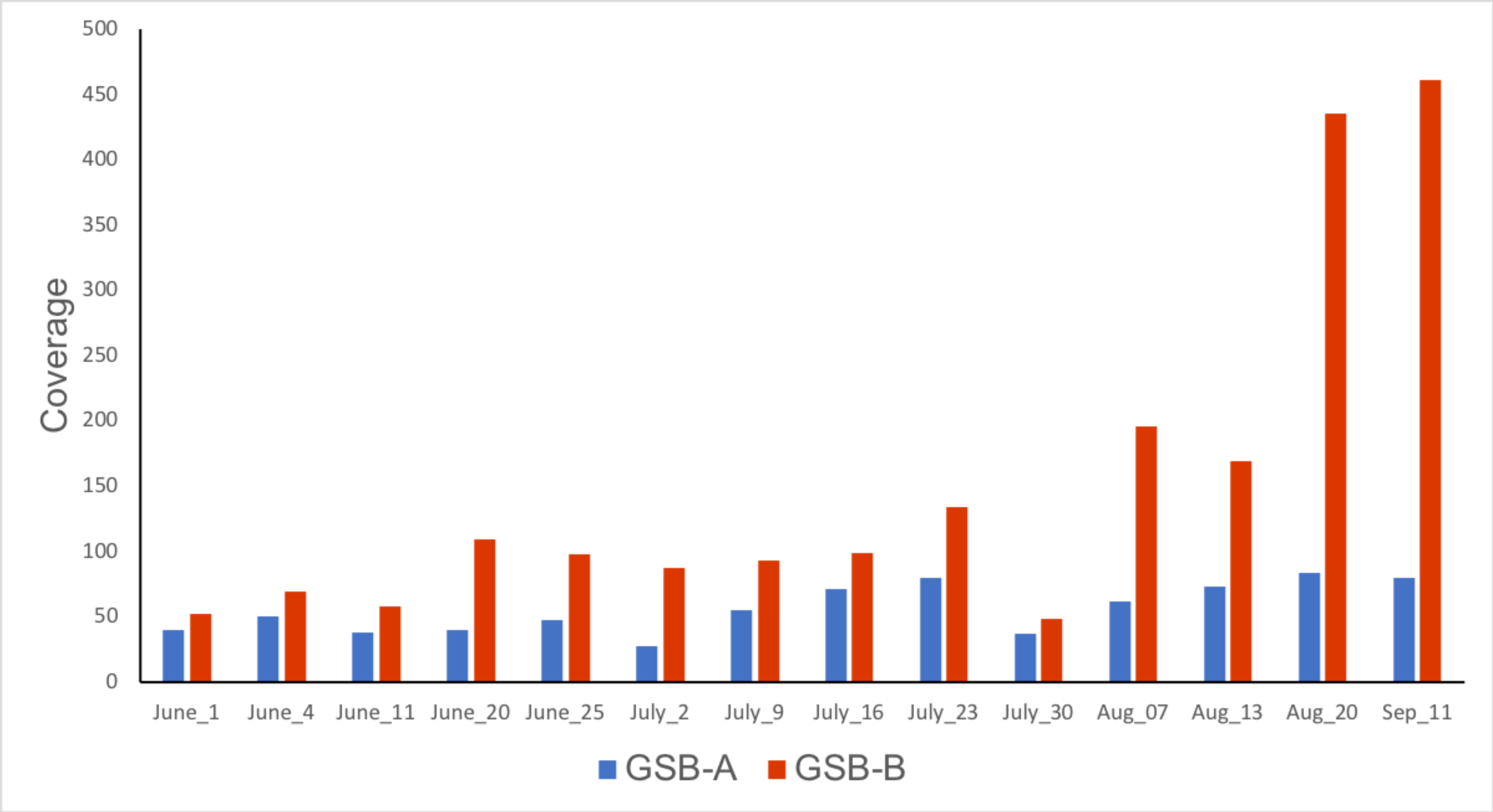

Figure S5. Shown are coverage values for GSB-A (blue, left bars) and GSB-B (red, right bars) for depth-integrated hypolimnion samples in 2018.

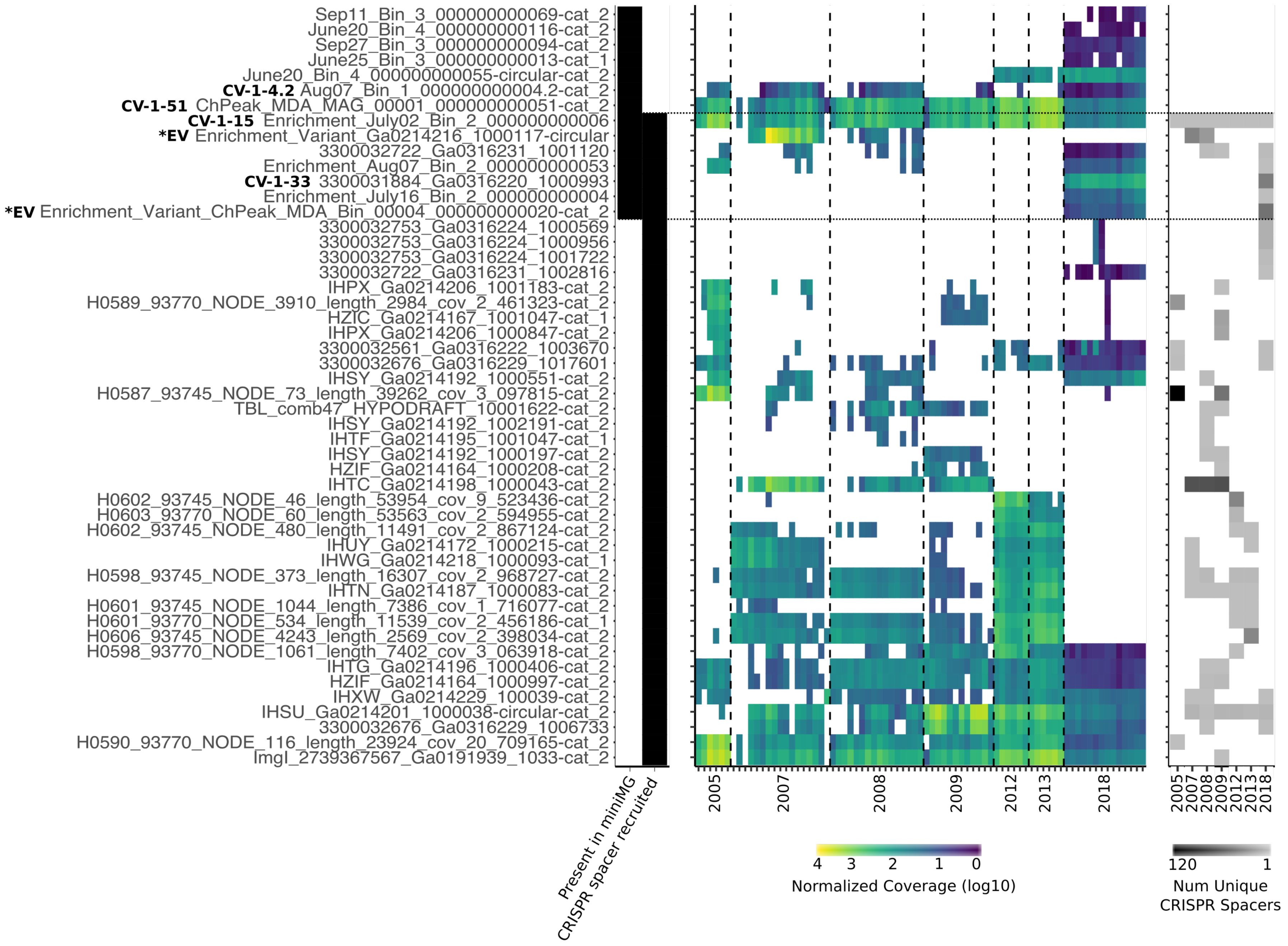

Figure S6. Shown are viral contigs identified through GSB CRISPR spacer matching, plus the identified GSB viral contigs. The four viral contigs discussed in this paper are labeled in bold; variants of CV-1-33 are labeled as *EV in bold. *(left)* Black to signify which contigs were found in the targeted metagenomes (miniMG), and which contigs were recruited through CRISPR spacer matching. *(center)* Normalized coverage for each sample across all contigs; white space represents insufficient or no coverage for that contig/sample. *(right)* Show are the number of unique CRISPR spacers acquired for each year.

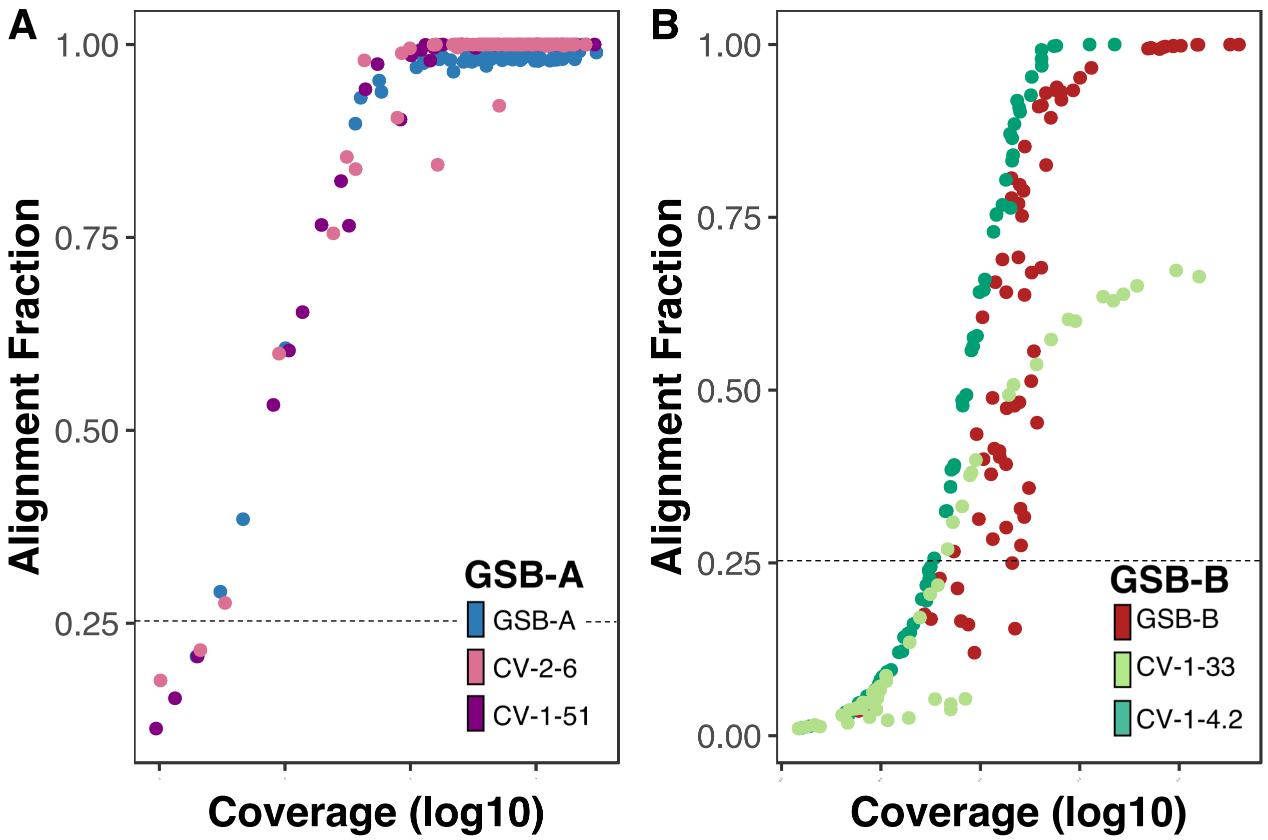

Figure S7. Raw coverage (log10) versus alignment fraction of GSB-A **(A)**, GSB-B **(B)**, and their associated viruses. CV-1-33 alignment fraction likely levels off around 65% due to the variable region between the different variants of this virus.

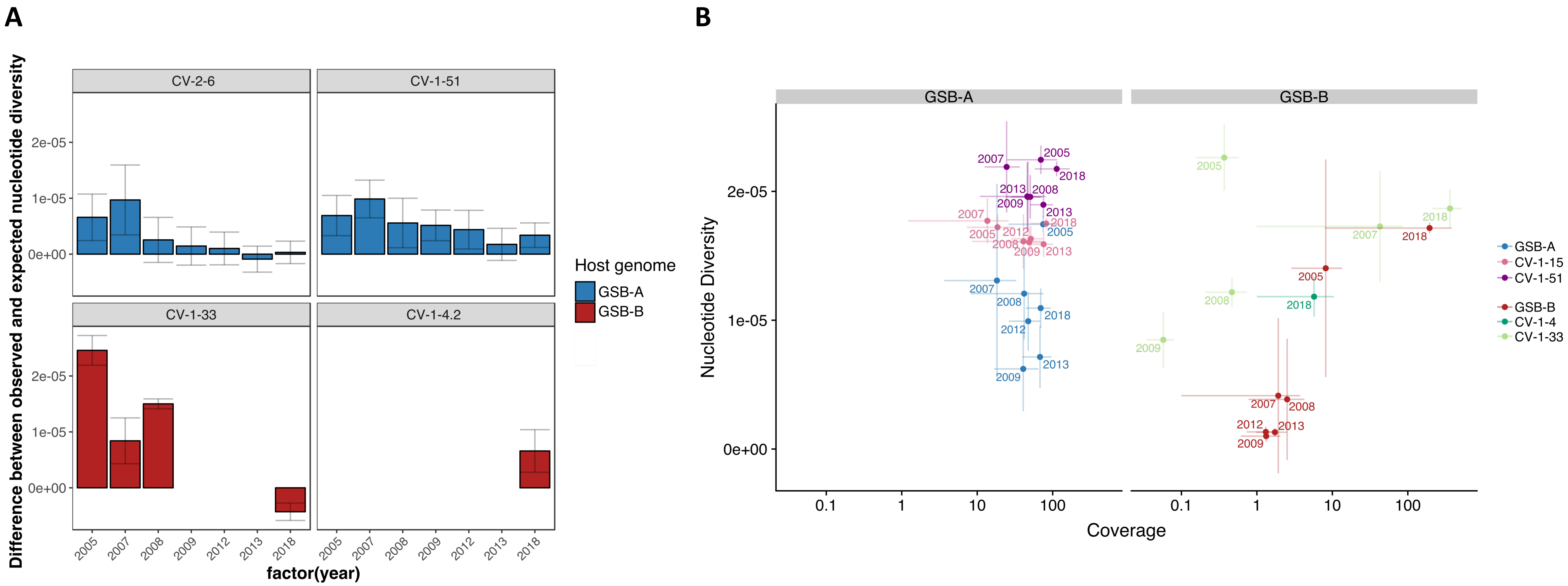

Figure S8. (A) Difference between observed and expected nucleotide diversity for GSB-A- and GSB-B-associated viruses; expected values are calculated using a regression of observed nucleotide diversity and raw (not normalized) coverage; bars show standard deviation. **(B)** Nucleotide diversity of GSB-A, GSB-B, and their associated viruses against coverage (log-scale; not normalized); bars show standard deviation.

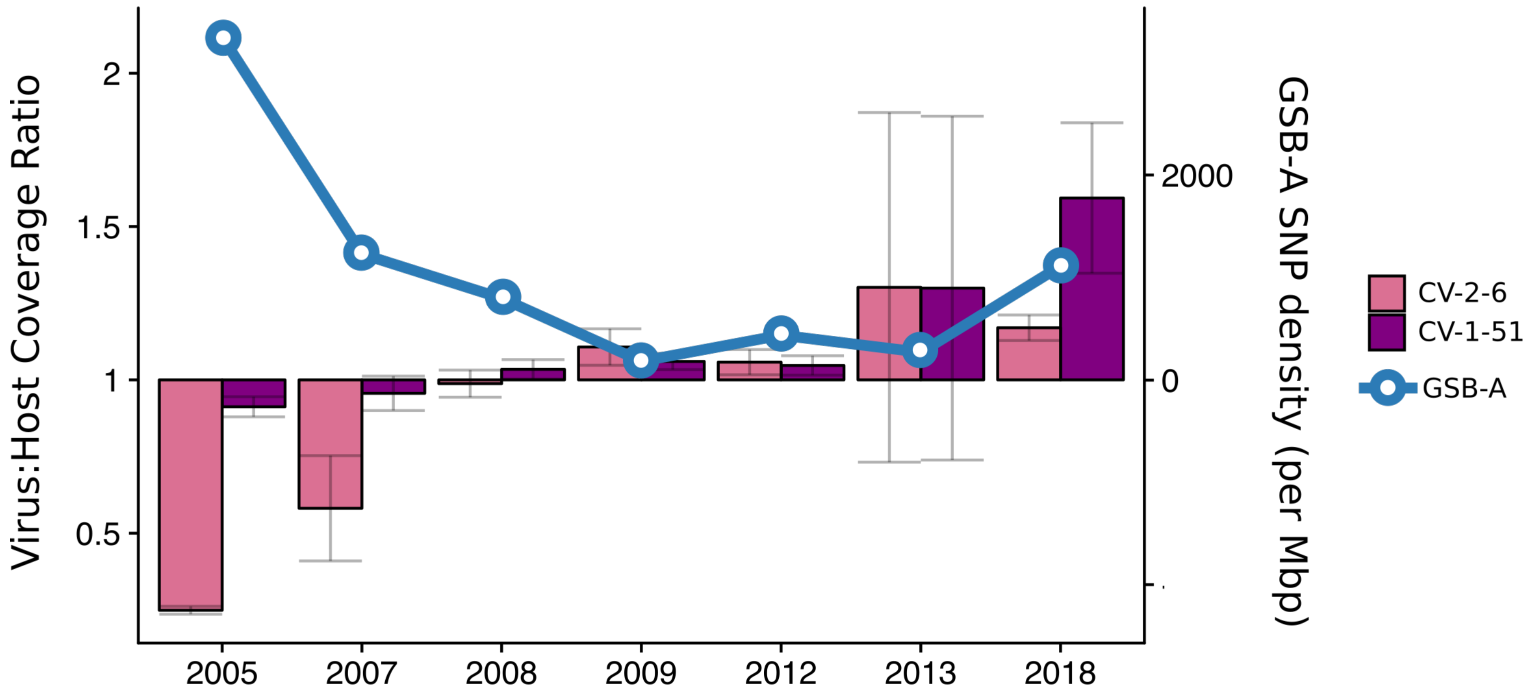

Figure S9. Shown are ratio of virus:host normalized coverage values of CV-2-6 and CV-1-51; error bars are standard deviation. Line represents the SNP density (SNPs per Mbp) for GSB-A.

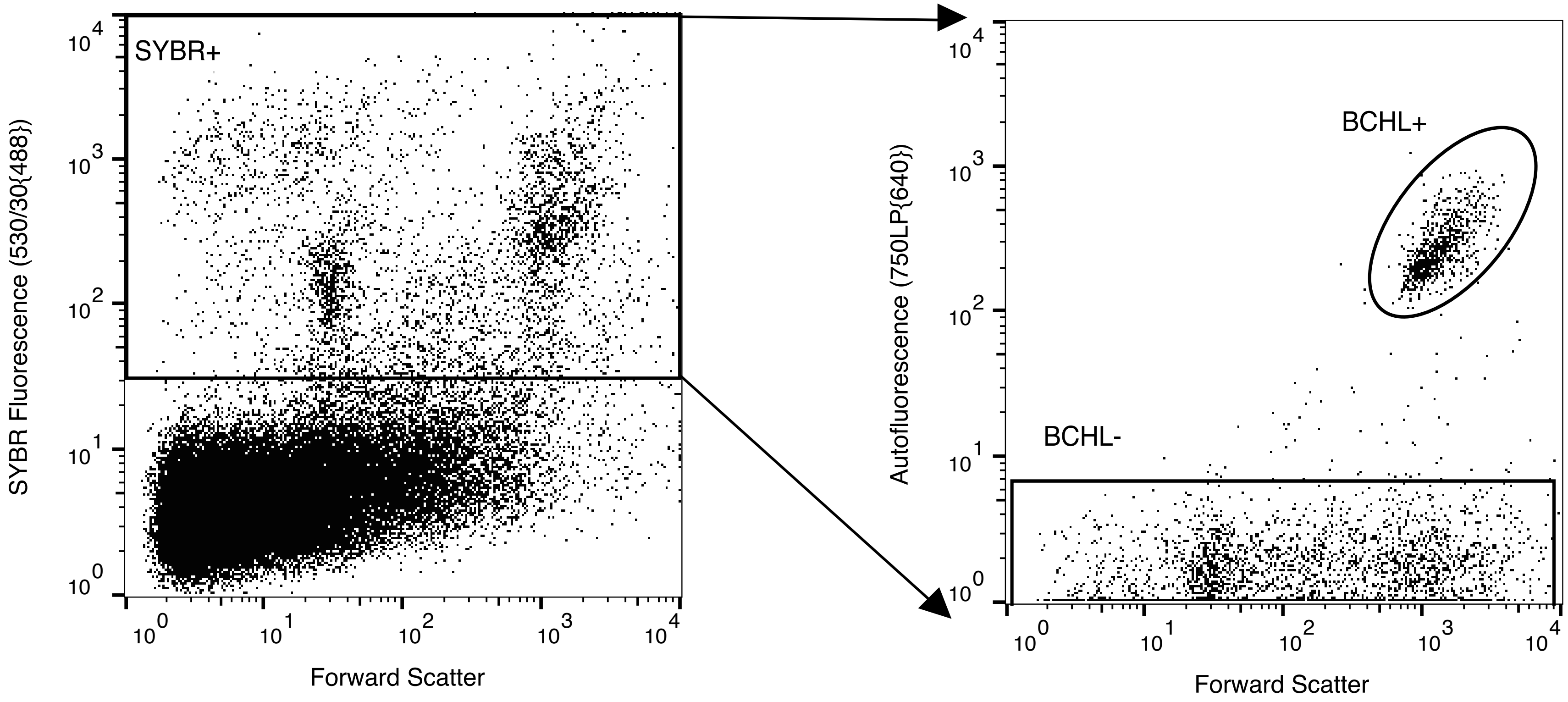

Figure S10. Illustration of flow cytometry signals and sort gates used for collecting GSBs. Panel A depicts how microbial cells were identified by labeling with generic DNA stain SYBR Green II and gating based on 530/40nm fluorescence when excited by a 488nm laser. The SYBR+ population was composed of a variety of cells types, including GSBs, with different sizes, DNA content, and other physical characteristics which led to the diversity of signals seen in the forward scatter and 530nm fluorescence channels. Events with fluorescence below the SYBR+ gate in panel A represent a mixture of instrument noise and unstained particles. Panel B focuses on only the SYBR+ cells and illustrates their fluorescence above 750nm when subsequently excited by a 640nm laser. The subset of SYBR+ cells that also contained bacteriochlorophyll (BCHL+) was distinguished by the presence of far-red autofluorescence in the 750LP channel when excited by the 640nm laser. Pictured above is a representative image from the 2.5m depth sampled on Aug 13, 2018. All samples contained at least one decade of separation on the 750LP channel between the BCHL+ and BCHL- gates used for flow sorting GSB and non-GSB cells, respectively.
